# Supplementary material for: Motivated misremembering of selfish decisions
Source: Nat Commun. 2020 Apr 29;11:2100. doi: 10.1038/s41467-020-15602-4 (PMC7190661; doi:10.1038/s41467-020-15602-4)
Supplement: Supplementary file 1 — Supplementary Information [file 41467_2020_15602_MOESM1_ESM.pdf]

Supplementary Information for

Motivated misremembering of selfish decisions

Ryan W. Carlson, Michel André Maréchal, Bastiaan Oud, Ernst Fehr, & Molly J. Crockett

Corresponding Authors: Molly J. Crockett & Ryan W. Carlson
Email: molly.crockett@yale.edu or ryan.carlson@yale.edu

This PDF file includes:

Supplementary Methods
Supplementary Results
Supplementary Discussion
Supplementary Figures 1 to 3 and Tables 1 to 15
Supplementary References

Supplementary Methods

Experiments 1 & 2: choice presentation format. In Experiments 1 and 2, choice options were presented in a graphical format for half the participants and a numeric format for the other half (See Supplementary Fig. 1). This manipulation was carried out to address a separate research question and its results are reported separately. The effect of choice format did not interact with any of the effects tested in the current experiments, thus, for simplicity, results are presented collapsed across choice format conditions.

Experiment 2: role knowledge manipulation. In Experiment 2, we also tested whether motivated misremembering is more likely to occur when people deviate from a self-serving versus impartial standard of fairness. In the absence of personal incentives – for instance, when people are in the role of a third-party observer, or are behind a veil of ignorance – people's beliefs about what is fair more closely reflect an impartial view[1]. However, research on self-serving biases has shown that personal incentives can strongly influence beliefs about fairness, inducing people to conflate what is fair with what is personally advantageous[2]. For example, in dictator games, beliefs about the size of a "fair" transfer are lower for dictators than receivers[1]. As such, here we additionally tested how such biases might influence the extent of misremembering among participants.

To address this question, participants were randomly assigned to one of two conditions. In the *full knowledge* condition, participants were informed that they were assigned to the role of Participant 1. Following this, participants were asked to indicate what they believed to be the "maximum acceptable share" for Participant 1 to keep. Finally, participants completed a series of five transfer decisions.

In the *veil of ignorance* condition, participants were informed that their role would be revealed to them at a later point in time. Following this, participants were asked to indicate what they believed to be the "maximum acceptable share" for Participant 1 to keep. Next, participants were informed that they were assigned to the role of Participant 1. Finally, participants completed a series of five transfer decisions.

Note that in the *full knowledge* condition, fairness beliefs were elicited *after* participants knew they would be the dictator and thus stood to profit from inequality. Thus, in this condition fairness beliefs reflect a combination of self-serving fairness beliefs and impartial fairness beliefs. Meanwhile, in the *veil of ignorance* condition, fairness beliefs were elicited *before* participants knew whether they would be the dictator or the recipient. Thus, in this condition fairness beliefs reflect impartial fairness beliefs only.

Experiment 3: **psychological consequences**. In Experiment 3, we additionally explored two factors that could shift as a function of whether an individual engaged in motivated misremembering or not: their affective state and their moral self-view.

Positively valenced affect. Specifically, we probed the extent to which each participant was experiencing positively valenced affect by asking (i) how *positive* they felt in the moment, and (ii) how *happy* they felt in moment—both on 7-point Likert scales ranging from 1 ("Not at all") to 7 ("Extremely"). These two items were closely related ($\alpha = .93$), and thus were averaged together to form our final measure of participants' positive affect.

Moral self-view. To probe each participant's moral self-view, we asked (i) to what extent they considered themselves a good person, and (ii) to what extent they considered themselves moral – again on 7-point Likert scales ranging from 1 ("Not at all") to 7 ("Extremely"). These two items were highly related ($\alpha = .89$), and thus were averaged together to form our final measure of participants' moral self-view.

Experiments 4a & 4b: **psychological consequences.** In Experiments 4a and 4b, we explored one additional factor that could shift as a function of whether an individual engaged in motivated misremembering or not: psychological discomfort.

We probed psychological discomfort at two time points; at the very beginning of the experiment (to provide a baseline), and after asking about memory confidence. We measured discomfort using a three-item scale[3], in which participants were asked to rate the extent to which they were currently feeling uncomfortable, uneasy, and bothered on a scale ranging from 1 ("Not at all") to 7 ("Extremely"). To avoid experimental demand[3], we randomly interleaved these discomfort items with five relatively valence-neutral items from the interest and activation subcomponents of positive affect (alert, attentive, determined, inspired, active)[4].

Supplementary Results

Experiment 1

Main results (with no exclusions, non-givers excluded, and static deciders excluded). To test the robustness of the findings in Experiment 1, we repeated our main analyses (i) while additionally excluding participants who never varied the amount they gave across their five transfer decisions (i.e., *static deciders*), (ii) while additionally excluding participants who never gave a positive amount across their five transfer decisions, and (iii) with no participants excluded. The first analyses are reported because individuals who made identical allocation decisions should have far less difficulty recalling how much they gave. As such, we wanted to ensure our results held when excluding these individuals—thereby creating more equal choice variance between groups (Supplementary Table 9). The second set of analyses are reported because individuals who never gave in our experiment were not expected to remember giving, nor could they possibly recall giving 'less' than they actually did. As such, we wanted to ensure these individuals did not bias our results in anyway. The third set of analyses were conducted simply to ensure our findings are robust to outliers. In all cases, the results were consistent with those reported in the main text. All analysis code is available at https://osf.io/pzwt7/

When including only those that varied in their decisions (N = 69), only those who gave a positive amount (N = 92), or when including all participants (N = 112), we again find that recalled generosity was significantly greater than actual generosity (Supplementary Table 10.1). Moreover, after performing a median split to group participants as behaviorally stingy and behaviorally generous, we again find that stingy participants remembered transferring significantly more than they actually did (Supplementary Table 10.2), whereas generous participants showed no such difference (Supplementary Table 10.3). In addition, stingy individuals trended towards making more self-serving memory errors than generous participants (Supplementary Table 10.4).

Memory inaccuracy. We tested if the two groups in Experiment 1 (stingy- and generous-behavior participants) differed in memory inaccuracy—or the absolute size of their memory errors. We found no significant difference between more stingy and more generous behaving participants in memory inaccuracy ($W = 1730.5$, $p = .12$, $d = .21$, $\delta = .17$; Supplementary Fig. 2a), and this result was consistent when using the three inclusion criteria assessed above (Supplementary Table 10.5).

Experiment 2

Main results (with no exclusions, non-givers excluded, and static deciders excluded). We also repeated our main analyses of Experiment 2 with static deciders excluded (N = 129), non-givers excluded (N = 184), as well as with no exclusions (N = 243). As with Experiment 1, the results were consistent with those reported in the main text.

We again found that participants showed a systematic bias towards self-serving memory errors, such that their recalled generosity was significantly greater than their actual generosity (Supplementary Table 11.1). In addition, we replicated the finding that specifically violators recalled being significantly more generous than they actually were (Supplementary Table 11.2), but not upholders (Supplementary Table 11.3). Moreover, when comparing violators and upholders directly, the former showed a significantly greater bias toward self-serving memory errors (Supplementary Table 11.4).

Moreover, when we perform a median split on upholders based on their generosity, we again find no significant difference between recalled generosity and actual generosity in either

stingy (Supplementary Table 11.6) nor generous upholders (Supplementary Table 11.7). We also found no differences between generous versus stingy upholders in self-serving memory errors (Supplementary Table 11.8).

Memory inaccuracy. Violators also showed greater inaccuracy than upholders, such that the absolute size of their memory errors tended to be larger ($W = 2787.5$, $p < .001$, $d = .70$, $\delta = .51$; Supplementary Fig. 2b). We also found that violators were less accurate overall than upholders when making no exclusions and when excluding non-givers (Supplementary Table 11.5). However, this effect was no longer significant when excluding static deciders ($p = .11$, $d = .26$, $\delta = .16$).

Role knowledge manipulation results. We examined whether our findings differed when making fairness judgments with full knowledge of one's assigned role, versus under a veil of ignorance. We found that participants' beliefs about what constitutes a fair offer were no different when made under a veil of ignorance compared with full knowledge ($W = 6198$, $p = .20$), nor were the variances in these beliefs ($\chi2_{\text{Fligner-Killeen}}$ (1) = .06, $p = .81$). These findings support the idea that our measure of personal standards was consistent and stable across conditions.

Participants' generosity was also not significantly affected by whether they were assigned to the full knowledge or veil of ignorance condition ($W = 5938$, $p = .078$). We also observed no differences in fairness deviations ($W = 6999.5$, $p = .75$) nor memory inaccuracy ($W = 6839$, $p = .99$) between conditions.

Norm deviation and choice variance in violators versus exceeders. Violators and exceeders were relatively well-matched in the extent to which they deviated from their norm (violators: $M = 13.65$, $SD = 10.81$; exceeders: $M = 11.28$, $SD = 9.52$; $W = 2091$, $p = .22$), and were somewhat similar in the extent to which their decisions varied, (violators: $M = 11.05$, $SD = 7.31$; exceeders: $M = 7.61$, $SD = 5.37$), however violators varied to a greater degree ($W = 1675.5$, $p = .003$).

Experiment 3

Main results (with no exclusions, non-givers excluded, and static deciders excluded). We also repeated our main analyses for Experiment 3 with static deciders excluded (N = 223), non-givers excluded (N = 504), as well as with no participants excluded (N = 647). As with Experiments 1 and 2, the results were consistent with those reported in the main text.

We replicated the finding that participants showed a systematic bias towards self-serving memory errors, such that their recalled generosity was significantly greater than their actual generosity (Supplementary Table 12.1). In addition, we replicated the finding that specifically violators recalled being significantly more generous than they actually were (Supplementary Table 12.2), but not upholders (Supplementary Table 12.3). Moreover, when comparing violators and upholders directly, the former showed a significantly greater bias toward self-serving memory errors (Supplementary Table 12.4).

In addition, when we group upholders based on a median split on their average generosity, we again find either small or non-significant differences between recalled generosity and actual generosity for stingy (Supplementary Table 12.6) and generous upholders (Supplementary Table 12.7). Consistent with our predictions, we find much smaller differences between generous- and stingy-behaving upholders in self-serving memory errors (Supplementary Table 12.8), compared to when participants were grouped based on whether they violated or upheld their fairness standards.

Memory inaccuracy. Violators also showed greater inaccuracy than upholders, such that the absolute size of their memory errors tended to be larger (W = 24440.5, p < .001, d = .55, δ = .26; Supplementary Fig. 2c). We also found that violators were less accurate overall than upholders when using the three inclusion criteria assessed above (Supplementary Table 12.5).

Ruling out a "confidence ratings as plausible deniability" account. Next, we assessed memory confidence. In Experiment 3, violators were significantly less confident in their recalled generosity than upholders (W = 39859.5, p < .001, d = .43, δ = .21).

Reporting lower memory confidence could reflect retrieval difficulty, but it could also reflect a strategy for maintaining plausible deniability if one were lying about their level generosity. To address this possibility, we tested whether confidence ratings were lower among violators who made *self-serving* versus *self-defeating* memory errors. A lying account would predict that confidence ratings should be lower specifically among those who made self-serving memory errors (i.e., those predicted to have a need to maintain plausible deniability). However, we found no significant difference in confidence ratings between violators who made self-serving memory errors (M = 4.86, SD = 1.83) versus self-defeating memory errors (M = 4.24, SD = 1.64; W = 349, p = .14, d = .35, δ = .23).

Norm deviation and choice variance in violators versus exceeders. Violators and exceeders were somewhat matched in the extent to which they deviated from their norm (violators: M = 23.61, SD = 17.43; exceeders: M = 18.11, SD = 13.59; W = 12499, p = .013, d= .34, δ = .16), though differed to a significant degree. In addition, they were well-matched in the degree to which their decisions varied (violators: M = 7.08, SD = 8.12; exceeders: M = 6.41, SD = 7.59; W = 14188, p = .50, d = .08, δ = .04).

Psychological consequences. One function of self-serving memory errors may be to reduce threats to one's moral self-image that can follow fairness violations. To test this hypothesis, we investigated whether moral self-views differed between violators and upholders as a function of whether their memory errors were self-serving (inflating their generosity) or self-defeating (deflating their generosity). As such, for these analyses we focused on participants who made memory errors (N = 160). First, we assessed how fairness standard compliance and memory error direction affected moral self-views. We found no main effect of violating (versus upholding) fairness standards ($H_{kruskal.wallis}(1)$ = .18, p = .67), nor exhibiting self-serving (vs. self-defeating) memory errors (H(1) = .14, p = .71) on participant's moral self-views. There was also no significant interaction between fairness standard compliance and memory error direction (H(3) = 2.71, p = .44) on moral self-views.

Another function of self-serving memory errors may be to counteract reductions in positive affect that may arise from violating one's standards of fairness. We specifically predicted that positive affect should be higher among violators who made self-serving (vs. self-defeating) memory errors. To test this, we examined whether positive affect differed specifically when violators made self-serving (versus self-defeating) memory errors. For violators, making self-serving (vs. self-defeating) memory errors significantly impacted positive affect (W = 301.5, p = .031, d = .56, δ = .33; Supplementary Fig. 3), such that violators who exhibited self-serving memory errors were happier than those who did not. However, no such shift in positive affect was observed among upholders (W = 1203.5, p = .68, d = .08, δ = .05).

Experiments 4a and 4b

Replicating findings from Experiments 2 and 3 in Experiment 4a. In addition to confirming the motive-dependent and choice-independent nature of misremembering, we also

sought to replicate our key analyses from Experiments 2 and 3 in Experiment 4a. To this end, we first examined memory errors in participants who gave *more* than what they indicated was fair (*exceeders*, N = 252). Consistent with prior experiments, we found that norm *exceeders* showed no evidence of a norm-directed memory errors, ($V = 4632$, $p = .96$, $d = .02$, $\delta = -.02$), and violators (N = 231) misremembered to a significantly greater degree than exceeders ($W = 22455$, $p < .001$, $d = .43$, $\delta = .23$).

Next, we compared the frequency of norm-directed memory errors among violators (N = 148) and exceeders (N = 136) who in fact made memory errors. A binomial test indicated that the proportion norm-directed memory errors made by violators was greater than chance ($p < 0.001$, 95% CI [.62, .77]), whereas the proportion of such errors made by exceeders was no different than chance ($p = .80$, 95% CI [.40, .57]).

When upholders were median split by their generosity into a behaviorally stingy group ($N = 121$) and a behaviorally generous group ($N = 124$), we again found no bias towards self-serving memory errors in either behaviorally stingy ($V = 1375.5$, $p = .52$, $d = .03$, $\delta = -.04$), nor generous upholders ($V = 865.5$, $p = .58$, $d = .06$, $\delta < .001$). Moreover, we also found no difference between stingy versus generous upholders in their tendency to make self-serving memory errors ($W = 7833$, $p = .53$, $d = .09$, $\delta = .04$).

As in prior experiments, we compared a model predicting recalled generosity from actual generosity, with one predicting recalled generosity from both actual generosity and the deviation between actual generosity and personal standards. In each model, we also controlled for choice speed, choice variance, non-giving, and numeracy. Replicating Experiments 2 & 3, in Experiment 4a we found that the model which additionally included fairness deviations again provided a superior fit (ΔAIC = 5.90, ΔBIC = 1.34, $\chi 2$ (1) = 7.90, $p = .005$; Supplementary Table 7). By contrast, in Experiment 4b (i.e., under forced-choice conditions), a comparison of these two models revealed that the model which additionally included fairness deviations did not provide a superior fit (ΔAIC = .87, ΔBIC = -3.49, $\chi 2$ (1) = 2.87, $p = .09$; See Supplementary Table 8).

Finally, as with our prior experiments, we find that our key findings for Experiments 4a (as well as Experiment 4b), remain consistent with no exclusions, with non-givers excluded, and with static deciders excluded (See Supplementary Tables 13, 14, and 15).

Memory inaccuracy. Here we examine whether those who freely violated their own standards show greater memory inaccuracy than those who were forced to violate their standards. Free choice violators showed greater inaccuracy than free choice upholders–making significantly larger memory errors ($W = 37086$, $p < .001$, $d = .41$, $\delta = .33$; Supplementary Fig. 2d). We also found that forced-choice violators showed greater inaccuracy than forced-choice upholders ($W = 31604.5$, $p < .001$, $d = .23$, $\delta = .20$), though this effect was smaller than that in the free choice condition.

Next, we assessed the role of responsibility in memory inaccuracy. To test this, we independently assessed the memories of those who reported being "not at all responsible" for their actions (i.e., 1 out of 7 on our personal responsibility measure; N=408), and those who self-reported some degree of personal responsibility for their actions (i.e., greater than 1 out of 7 on our personal responsibility measure; M = 4.87, SD = 1.84; N = 171). We found that violators showed greater inaccuracy than upholders—making significantly larger memory errors—both when they felt responsible for their choices ($W = 2778$, $p = .006$, $d = .32$, $\delta = .24$), and when they did not feel responsible ($W = 16013.5$, $p = .006$, $d = .01$, $\delta = .14$).

Next, we examined the subset of Experiment 4a free-choice deciders who were yoked with Experiment 4b forced-choice deciders (N = 580), we found that free-choice violators showed greater inaccuracy than upholders–making significantly larger memory errors (W = 23396.5, p < .001, d = .43, δ = .35). Similarly, yoked forced-choice deciders—who were forced to make, and then remember, the exact same choice sets as free-choice deciders—also showed this effect, such that those paired with violators (vs upholders) tended to have less accurate memories in general (W = 23396.5, p < .001, d = .43, δ = .35). However, as mentioned further down in the supplementary results, memory inaccuracy in forced-choice violators did not predict self-serving memories in yoked free-choice violators.

Ruling out a "confidence ratings as plausible deniability" account in Experiment 4a. Consistent with Experiment 3, violators were significantly less confident in their recalled generosity than upholders (W = 69371.5, p < .001, d = .42, δ = .26).

As mentioned in the supplementary results for Experiment 3, reporting lower memory confidence could reflect retrieval difficulty, or a strategy for maintaining plausible deniability if one were lying about their level generosity. To rule out the plausible deniability account, we tested whether confidence ratings were lower among violators who made *self-serving* versus *self-defeating* memory errors. A lying account would predict that confidence ratings should be lower specifically among those who made self-serving memory errors (i.e., those predicted to have a need to maintain plausible deniability). However, we found no significant difference in confidence ratings between violators who made self-serving memory errors versus self-defeating memory errors (W = 2176, p = .63, d = .07, δ = .05).

Norm deviation and choice variance in violators versus exceeders. In Experiment 4a, violators and exceeders were matched in the extent to which they deviated from their norm (violators: M = 16.57, SD = 15.98; exceeders: M = 14.29, SD = 12.32; W = 28579, p = .73, d = .16, δ = .02. In addition, they were matched in the degree to which their decisions varied (violators: M = 8.30, SD = 7.67; exceeders: M = 6.90, SD = 6.29; W = 26336, p = .067, d = .20, δ = .10)

Psychological consequences in Experiment 4a. Next, we investigated whether self-reported psychological discomfort differed between violators and upholders as a function of whether their memory errors were self-serving (inflating their generosity) or self-defeating (deflating their generosity). As such, for these analyses we focused on participants who made memory errors (N = 305). First, we assessed how fairness standard compliance and memory error direction affected discomfort. To measure this, we took the difference between discomfort reported at the beginning of the experiment, and discomfort reported after the recall stage. We found no main effect of violating (vs. upholding) fairness standards ($H_{kruskal.wallis}(1) = 2.03$, p = .15), nor exhibiting self-serving (vs. self-defeating) memory errors (H(1) = .77, p = .38) on participant's psychological discomfort. This suggests that on average, violators did not experience more discomfort than upholders, nor did self-serving memory errors lead to less discomfort than self-defeating memory errors. Interestingly, there was also no significant interaction between fairness standard compliance and memory error direction (H(3) = 1.45, p = .69) on discomfort, suggesting that violators who made self-serving memory errors did not experience less discomfort than those who made self-defeating memory errors.

That violators experienced no more discomfort than upholders at recall, while showing a substantially greater degree of self-serving memory errors, is consistent with a dissonance reduction account. Though this same account would also have predicted that self-defeating errors would increase discomfort, which was not observed. Of course, interpreting self-reported

discomfort is complicated by the reality that people may adopt numerous strategies to relieve dissonance[5], and people tend to take the first path that they perceive to be available for reducing dissonance[6]. Some violators could have plausibly convinced themselves after the fact that their actions were not representative of their true self. Such a strategy could effectively relieve the felt discrepancy between one's actions and their morals, and render self-defeating memory errors inconsequential.

A further test of the choice independence of misremembering: does memory inaccuracy among forced-choice violators (Experiment 4b) predict self-serving memory errors in free-choice violators (Experiment 4a)? Since forced-choice violators showed greater memory inaccuracy than upholders—just as free choice violators did—it is possible that the memory errors of forced-choice violators might reflect the 'true' memorability of free violators choices, and that this true level of memorability could be related to free choice violators tendency to make self-serving memory errors. A reviewer suggested that it is important to rule out this possibility, as the same cause of memory inaccuracy in forced-choice violators could be related to self-serving memory errors in yoked, free-choice violators.

That is, among those free-choice deciders who made memory errors, can the direction of their errors (self-serving versus self-defeating) be predicted by memory inaccuracy in yoked forced-choice deciders? More importantly, does it have predictive value above and beyond whether or not a free-choice decider violated their norm?

We tested this possibility with a logistic regression model in which memory error direction (self-serving [1] vs. self-defeating [0]) was predicted by (i) norm violation status (violated [1] vs. upheld [0]), (ii) memory inaccuracy of yoked forced-choice deciders, and the interaction of (i) and (ii). The results supported the choice-independence account: while the model explained a significant degree of the variance ($R^2 = .036$, $F(3,241) = 3.00$, $p = .031$), the only significant predictor was the free-choice decider's norm violation status ($\beta = .083$, $p < .004$). Indeed, neither the memory errors of yoked forced-choice deciders ($\beta = -.020$, $p = .57$), nor the interaction of norm violation status and these yoked memory errors ($\beta = .011$, $p = .73$), significantly predicted the occurrence of self-serving memory errors in free-choice deciders.

Supplementary Discussion

Note on choice variability and memorability. Above we show that our findings remain consistent when only including those who made dynamic decisions (i.e., when we exclude static deciders). This supports the idea that motivated misremembering cannot be simply explained by choice set memorability. This possibility seemed plausible given that violators showed greater memory inaccuracy. Yet whereas excluding static deciders—and thus balancing the choice variance gap between groups—greatly diminishes memory inaccuracy differences between violators and upholders, the motivated misremembering difference remains the same. In addition, motivated misremembering among violators disappears when personal responsibility is removed, which has a negligible influence on memory inaccuracy. This pattern of results is consistent with the view that group differences in choice variance drive memory inaccuracy differences, but they do not drive motivated misremembering. Further support for the idea that motivated misremembering is choice independent was garnered by demonstrating that memory errors among forced-choice deciders do not predict self-serving memory errors in free-choice deciders.

Of course, one question that is relevant to our research is why participants would make different decisions across the five different rounds. We offer three explanations for why we expected this to occur. First, people tend to have uncertainty about their preferences, which would manifest through choice variance[7]. Moreover, even when one's preferences are certain, the cognitive and neural mechanisms that translate preferences into choices tend to be somewhat noisy[8]. Finally, each allocation decision could be influenced to some extent by thoughts and feelings experienced after one's previous allocation decision (e.g., satisfaction felt after making a fair decision may make people less moral on the next trial, or guilt experienced after an unfair decision may lead people to make a more generous distribution on the next trial[9,10]. As such, we expected people to exhibit variability even when facing the same choice repeatedly.

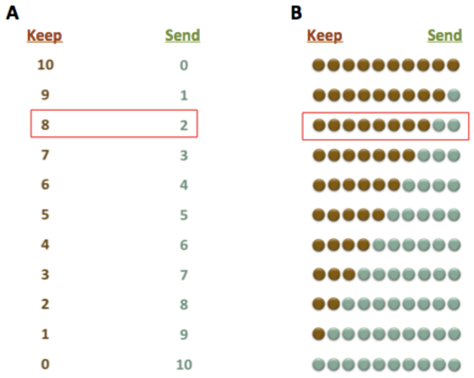

Supplementary Fig. 1. Choice format. Participants were asked to decide what percentage of the stake they would like to keep for themselves and what percentage of the stake they would like to send to the receiver. In the numeric format (**a**), each point represented 10% of the stake. In the graphical format (**b**), each ball in the diagram represented 10% of the stake. Experiments 1 and 2 were run in German; the figure depicts the English translation.

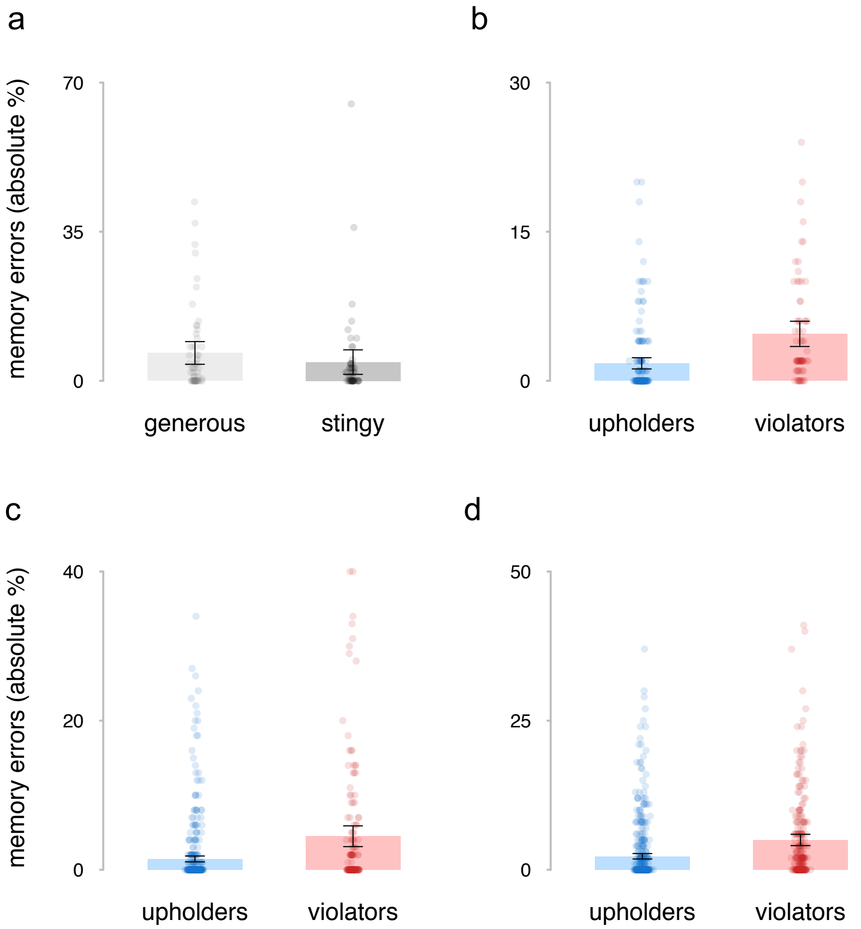

Supplementary Fig. 2. Here we show absolute differences between the mean percent participant's recalled giving, versus how much they actually gave. Error bars represent 95% CIs. **(a)** In Experiment 1, stingy- versus generous-behaving participants did not significantly differ in the extent of their memory inaccuracy. **(b)** In Experiment 2, however, we find that those violating their own personal standard of fairness (violators) showed greater memory inaccuracy—making larger memory errors than those upholding their personal standard of fairness (upholders). In Experiment 3 **(c)** and Experiment 4a **(d)**, we again find that violators showed greater memory inaccuracy than upholders.

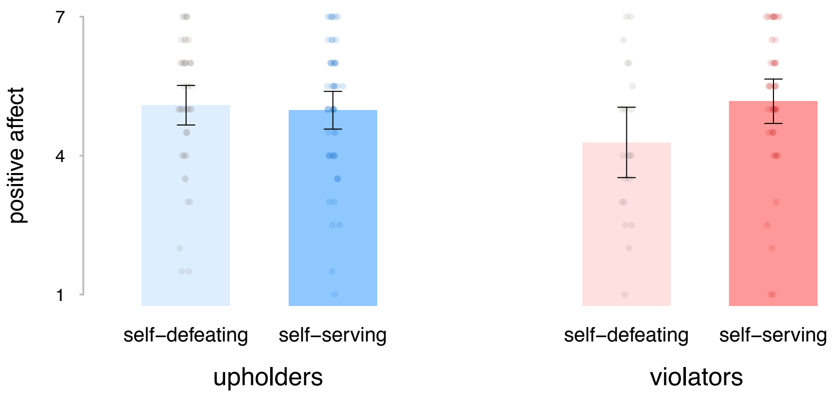

Supplementary Fig. 3. Violators who made self-serving memory errors reported greater positive affect than those who made self-defeating memory errors. Crucially, this difference was not observed among upholders. Error bars reflect 95% CIs.

Supplementary Table 1. Descriptive statistics for key comparisons across all five experiments.

	actual giving		recalled giving	
	M	*SD*	*M*	*SD*
Experiment 1				
all participants (N = 109)	26.68	18.74	29.24	20.76
stingy (N = 53)	10.11	9.55	13.75	16.14
generous (N = 56)	42.36	9.40	43.90	12.26
Experiment 2				
all participants (N = 234)	23.32	18.11	24.37	18.15
violators (N = 69)	22.29	13.29	25.65	13.86
upholders (N = 165)	23.75	19.81	23.83	19.68
Experiment 3				
all participants (N = 604)	31.21	20.71	32.00	20.88
violators (N = 143)	15.97	16.65	18.72	19.14
upholders (N = 461)	35.94	19.55	36.13	19.66
Experiment 4a				
all participants (N = 709)	36.72	23.39	37.89	23.85
violators (N = 231)	28.88	23.56	32.20	25.79
upholders (N = 478)	40.51	22.36	40.64	22.37
Experiment 4b				
all participants (N = 579)	37.02	23.10	38.01	22.84
violators (N = 217)	20.95	18.25	23.87	19.57
upholders (N = 362)	46.65	20.16	46.49	20.33
Experiment 4b – no resp.				
all participants (N = 408)	36.89	22.93	37.07	22.85
violators (N = 138)	20.00	17.49	20.22	17.26
upholders (N = 270)	45.53	20.46	45.68	20.45
Experiment 4b – resp.				
all participants (N = 171)	37.31	23.55	40.26	22.72
violators (N = 79)	22.61	19.52	30.23	21.74
upholders (N = 92)	49.93	19.00	48.88	19.92

Supplementary Table 2. Descriptive statistics for further comparisons across all five experiments.

	actual giving		recalled giving	
	M	*SD*	*M*	*SD*
Experiment 2				
upholders - stingy (N = 82)	6.34	8.73	6.62	8.53
upholders - generous (N = 83)	40.94	10.36	40.84	10.66
violators (N = 69)	22.29	13.29	25.65	13.86
exceeders (N = 69)	30.84	15.17	30.85	15.08
Experiment 3				
upholders - stingy (N = 230)	20.36	15.30	21.08	16.12
upholders - generous (N = 231)	51.45	6.81	51.11	7.90
violators (N = 143)	15.97	16.65	18.72	19.14
exceeders (N = 207)	38.25	15.58	38.47	15.85
Experiment 4a				
upholders - stingy (N = 229)	23.02	16.13	23.45	16.98
upholders - generous (N = 249)	56.59	13.43	56.46	13.16
violators (N = 231)	28.88	23.56	32.20	25.79
exceeders (N = 252)	45.01	20.48	45.11	20.98
Experiment 4b (N=579)				
upholders - stingy (N = 133)	27.49	14.28	28.92	17.04
upholders - generous (N = 229)	57.77	13.69	56.70	14.21
violators (N = 217)	20.95	18.25	23.87	19.57
exceeders (N = 276)	48.93	19.90	48.04	19.91
Experiment 4b – no resp.				
upholders - stingy (N = 102)	26.27	15.16	27.39	16.54
upholders - generous (N = 168)	57.21	13.09	56.79	13.40
violators (N = 138)	20.00	17.49	20.22	17.26
exceeders (N = 204)	48.05	19.77	47.63	19.64
Experiment 4b – resp.				
upholders - stingy (N = 31)	31.48	10.07	33.97	17.96
upholders - generous (N = 61)	59.31	15.23	56.46	16.35
violators (N = 79)	22.61	19.52	30.23	21.74
exceeders (N = 72)	51.44	20.22	49.22	20.75

Supplementary Table 3. Correlations between memory inaccuracy and control variables across all five experiments.

		memory inaccuracy
Experiment 1	choice variance	.69**
	choice speed	.28**
	non-giving	.39**
	numeracy	-.07
Experiment 2	choice variance	.71**
	choice speed	.21**
	non-giving	.35**
	numeracy	-.30**
Experiment 3	choice variance	.69**
	choice speed	.18**
	non-giving	.25**
	numeracy	-.35**
	social desirability	.10*
Experiment 4a	choice variance	.63**
	choice speed	.26**
	non-giving	.28**
	numeracy	-.50**
Experiment 4b	choice variance	.57**
	choice speed	.12**
	non-giving	.21**
	numeracy	-.28*

Note. Correlations were calculated using Spearman's rho. * indicates $p < .05$. ** indicates $p < .01$.

Supplementary Table 4. Group comparisons on control variables across all experiments.

		W	*p*	*d*	δ
Experiment 1: stingy vs. generous					
	choice variance	1651.5	.30	.34	.11
	choice speed	1603	.28	.13	.12
	non-giving	1960	<.001	.95	.32
	numeracy	1382.5	.50	.26	.07
Experiment 2: violators vs. upholders					
	choice variance	2037	<.001	1.30	.64
	choice speed	4862	.08	.09	.15
	non-giving	4318.5	<.001	.61	.24
	numeracy	7148.5	<.001	.52	.26
Experiment 3: violators vs. upholders					
	choice variance	22090.5	<.001	.58	.33
	choice speed	33735.5	.67	.14	.02
	non-giving	39401.5	<.001	.54	.20
	numeracy	37970	.01	.17	.15
	social desirability	30875	.25	.11	.06
Experiment 4a: violators vs. upholders					
	choice variance	34300	<.001	.59	.38
	choice speed	49028	.02	.15	.11
	non-giving	57189.5	.15	.11	.04
	numeracy	68427.5	<.001	.37	0.24
Experiment 4b: violators vs. upholders					
	choice variance	32522	<.001	.21	.17
	choice speed	35201.5	.04	.24	.10
	non-giving	46446	<.001	.61	.18
	numeracy	43271	.04	.17	.10

Note. Group differences were calculated using Mann-Whitney U-tests.

Supplementary Table 5. Model comparison for Experiment 2 using recalled giving as the criterion.

Predictor	*b*	*beta*	*beta* 95% CI	sr^2	*r*	Fit	Difference
(Intercept)	5.46						
actual giving	.94**	.94	[.89, .98]	.47	.96**		
choice speed	.00	.01	[-.02, .05]	.00	.13*		
choice variance	.06	.02	[-.02, .06]	.00	.25**		
non-giving	1.18	.03	[-.03, .08]	.00	.67**		
numeracy	-5.01	-.03	[-.07, .01]	.00	-.08		
						R^2 = .927** 95% CI[.91,.94]	
(Intercept)	3.71						
actual giving	.96**	.96	[.91, 1.01]	.46	.96**		
choice speed	.00	.02	[-.01, .06]	.00	.13*		
choice variance	.01	.00	[-.04, .04]	.00	.25**		
non-giving	.88	.02	[-.03, .07]	.00	.67**		
numeracy	-3.53	-.02	[-.06, .01]	.00	-.08		
fairness deviation	.10**	.07	[.03, .11]	.00	-.14*		
						R^2 = .932** 95% CI[.91,.94]	ΔR^2 = .004** 95% CI[-.00, .01]

Note. Models are Ordinary Least Squares (OLS) regressions. A significant *b*-weight indicates the beta-weight and semi-partial correlation are also significant. *b* represents unstandardized regression weights. *beta* indicates the standardized regression weights. sr^2 represents the semi-partial correlation squared. *r* represents the zero-order correlation. *LL* and *UL* indicate the lower and upper limits of a confidence interval, respectively.
* indicates $p < .05$. ** indicates $p < .01$.

Supplementary Table 6. Model comparison for Experiment 3 using recalled giving as the criterion.

Predictor	*b*	*beta*	*beta* 95% CI	sr^2	*r*	Fit	Difference
(Intercept)	1.61						
actual giving	.93**	.92	[.89, .95]	.44	.96**		
choice speed	.12**	.03	[.01, .06]	.00	.03		
choice variance	.03	.01	[-.01, .03]	.00	.07		
non-giving	2.99**	.05	[.02, .09]	.00	.68**		
numeracy	-.18	-.02	[-.04, .01]	.00	-.06		
social desirability	.02	.00	[-.02, .03]	.00	.11**		
						R^2 = .921**	
						95% CI[.91,.93]	
(Intercept)	1.00						
actual giving	.95**	.94	[.90, .97]	.41	.96**		
choice speed	.11*	.03	[.01, .05]	.00	.03		
choice variance	.03	.01	[-.01, .04]	.00	.07		
non-giving	3.12**	.06	[.02, .09]	.00	.68**		
numeracy	-.17	-.02	[-.04, .01]	.00	-.06		
social desirability	.01	.00	[-.02, .02]	.00	.11**		
fairness deviation	.04**	.04	[.01, .06]	.00	-.43**		
						R^2 = .922**	ΔR^2 = .001**
						95% CI[.91,.93]	95% CI[-.00, .00]

Note. Models are OLS regressions. A significant *b*-weight indicates the beta-weight and semi-partial correlation are also significant. *b* represents unstandardized regression weights. *beta* indicates the standardized regression weights. sr^2 represents the semi-partial correlation squared. *r* represents the zero-order correlation. *LL* and *UL* indicate the lower and upper limits of a confidence interval, respectively.
* indicates $p < .05$. ** indicates $p < .01$.

Supplementary Table 7. Model comparison for Experiment 4a using recalled giving as the criterion.

Predictor	*b*	*beta*	*beta* 95% CI	sr^2	*r*	Fit	Difference
(Intercept)	4.92**						
actual giving	.94**	.92	[.90, .95]	.51	.96**		
choice speed	-.02	-.01	[-.03, .01]	.00	.11**		
choice variance	.03	.01	[-.01, .03]	.00	.08*		
non-giving	2.65**	.03	[.01, .06]	.00	.55**		
numeracy	-.38**	-.05	[-.07, -.03]	.00	-.37**		
						R^2 = .925**<br>95% CI[.92,.93]	
(Intercept)	3.83**						
actual giving	.95**	.94	[.91, .96]	.48	.96**		
choice speed	-.02	-.01	[-.03, .01]	.00	.11**		
choice variance	.02	.01	[-.02, .03]	.00	.08*		
non-giving	2.88**	.04	[.01, .06]	.00	.55**		
numeracy	-.33**	-.04	[-.07, -.02]	.00	-.37**		
fairness deviation	.04**	.03	[.01, .05]	.00	-.31**		
						R^2 = .925**<br>95% CI[.92,.93]	ΔR^2 = .001**<br>95% CI[-.00, .00]

Note. Models are OLS regressions. A significant *b*-weight indicates the beta-weight and semi-partial correlation are also significant. *b* represents unstandardized regression weights. *beta* indicates the standardized regression weights. sr^2 represents the semi-partial correlation squared. *r* represents the zero-order correlation. *LL* and *UL* indicate the lower and upper limits of a confidence interval, respectively.
* indicates $p < .05$. ** indicates $p < .01$.

Supplementary Table 8. Model comparison for Experiment 4b (no responsibility) using recalled giving as the criterion.

Predictor	*b*	*beta*	*beta* 95% CI	sr^2	*r*	Fit	Difference
(Intercept)	1.43						
actual giving	.96**	.96	[.93, .99]	.61	.97**		
choice speed	.10	.01	[-.01, .04]	.00	-.13**		
choice variance	.03	.01	[-.01, .03]	.00	.10*		
non-giving	1.86	.03	[-.00, .05]	.00	.58**		
numeracy	-.17	-.01	[-.04, .01]	.00	.02		
						R^2 = .946**<br>95% CI[.94,.95]	
(Intercept)	1.61						
actual giving	.95**	.95	[.92, .99]	.36	.97**		
choice speed	.10	.01	[-.01, .04]	.00	-.13**		
choice variance	.03	.01	[-.01, .03]	.00	.10*		
non-giving	1.88	.03	[-.00, .06]	.00	.58**		
numeracy	-.17	-.01	[-.04, .01]	.00	.02		
fairness deviation	-.01	-.01	[-.04, .02]	.00	-.68**		
						R^2 = .946**	ΔR^2 = .000

Note. Models are OLS regressions. A significant *b*-weight indicates the beta-weight and semi-partial correlation are also significant. *b* represents unstandardized regression weights. *beta* indicates the standardized regression weights. sr^2 represents the semi-partial correlation squared. *r* represents the zero-order correlation. *LL* and *UL* indicate the lower and upper limits of a confidence interval, respectively.

* indicates $p < .05$. ** indicates $p < .01$.

Supplementary Table 9. Group comparison (violators versus upholders) on choice variance with static deciders excluded.

	W	*p*	*d*	**δ**
Exp. 2	1462	.003	.55	.30
Exp. 3	5700.5	.61	.09	.04
Exp. 4a	17980	.64	.13	.03
Exp. 4b (r)	1135.5	.93	.12	.01
Exp. 4b (nr)	5518.5	.97	.13	.003

Note. Group differences were calculated using Mann-Whitney U-tests. Responsibility = (r), No responsibility = (nr)

Supplementary Table 10. Experiment 1 results (with no exclusions, non-givers excluded, and static deciders excluded)

	test statistic	*p*	*d*	**δ**
No exclusions (N=112)				
1. recalled vs. actual giving (all)	1474.5	.018	.17	.14
2. recalled vs. actual giving (stingy)	394.5	< .001	.35	.25
3. recalled vs. actual giving (generous)	353	.76	.01	.04
4. recalled vs. actual giving (s > g)	1292.5	.098	.38	.18
5. memory inaccuracy (s > g)	1785.5	.19	.05	.14
Non-givers excluded (N=92)				
1. recalled vs. actual giving (all)	1288	.029	.24	.15
2. recalled vs. actual giving (stingy)	394.5	.004	.40	.31
3. recalled vs. actual giving (generous)	264	.76	.09	.02
4. recalled vs. actual giving (s > g)	820	.067	.35	.22
5. memory inaccuracy (s > g)	991.5	.64	.07	.06
Static deciders excluded (N=69)				
1. recalled vs. actual giving (all)	1268.5	.020	.28	.22
2. recalled vs. actual giving (stingy)	307.5	.004	.43	.34
3. recalled vs. actual giving (generous)	342	.45	.17	.11
4. recalled vs. actual giving (s > g)	506.5	.31	.23	.14
5. memory inaccuracy (s > g)	739.5	.076	.24	.25

Note. Within-group tests and between-group tests were calculated using Wilcoxon sign-ranked tests and Mann-Whitney U-tests, respectively.

Supplementary Table 11. Experiment 2 results (with no exclusions, non-givers excluded, and static deciders excluded)

	test statistic	*p*	*d*	δ
No exclusions (N=243)				
1. recalled vs. actual giving (all)	4663	.019	.10	.10
2. recalled vs. actual giving (violators)	1399	< .001	.46	.36
3. recalled vs. actual giving (upholders)	938	.63	.05	-.01
4. recalled vs. actual giving (v > u)	4049	< .001	.52	.33
5. memory inaccuracy (v > u)	3115	< .001	.43	.49
6. recalled vs. actual giving (stingy upholders)	132	.32	.12	.05
7. recalled vs. actual giving (gen. upholders)	357	.16	.16	-.06
8. recalled vs. actual giving (s.u > g.u)	3376	.20	.29	.10
Non-givers excluded (N=184)				
1. recalled vs. actual giving (all)	3850	.017	.20	.11
2. recalled vs. actual giving (violators)	1289.5	< .001	.53	.36
3. recalled vs. actual giving (upholders)	647	.41	.03	-.03
4. recalled vs. actual giving (v > u)	2509.5	< .001	.65	.36
5. memory inaccuracy (v > u)	2241	< .001	.55	.42
6. recalled vs. actual giving (stingy upholders)	195.5	.88	.06	.02
7. recalled vs. actual giving (gen. upholders)	130	.16	.16	-.08
8. recalled vs. actual giving (s.u > g.u)	1580	.36	.18	.09
Static deciders excluded (N=129)				
1. recalled vs. actual giving (all)	3742	.014	.26	.16
2. recalled vs. actual giving (violators)	1289.5	< .001	.54	.38
3. recalled vs. actual giving (upholders)	595	.39	.02	-.06
4. recalled vs. actual giving (v > u)	1321	< .001	.58	.36
5. memory inaccuracy (v > u)	1748.5	.11	.26	.16
6. recalled vs. actual giving (stingy upholders)	118	.79	.03	<.001
7. recalled vs. actual giving (gen. upholders)	190.5	.39	.05	-.11
8. recalled vs. actual giving (s.u > g.u)	479	.61	.08	.08

Supplementary Table 12. Experiment 3 results (with no exclusions, non-givers excluded, and static deciders excluded)

	test statistic	*p*	*d*	**δ**
No exclusions (N=647)				
1. recalled vs. actual giving (all)	11047	< .001	.16	.06
2. recalled vs. actual giving (violators)	2466.5	< .001	.34	.18
3. recalled vs. actual giving (upholders)	2995.5	.39	.07	.02
4. recalled vs. actual giving (v > u)	33184.5	< .001	.39	.16
5. memory inaccuracy (v > u)	27948.5	< .001	.46	.29
6. recalled vs. actual giving (stingy upholders)	1645	.018	.19	.07
7. recalled vs. actual giving (gen. upholders)	188	.062	.11	-.03
8. recalled vs. actual giving (s.u > g.u)	26471.5	.011	.31	.10
Non-givers excluded (N=504)				
1. recalled vs. actual giving (all)	7835	.011	.14	.05
2. recalled vs. actual giving (violators)	1558.5	< .001	.37	.22
3. recalled vs. actual giving (upholders)	2346.5	.81	.04	.01
4. recalled vs. actual giving (v > u)	15741.5	< .001	.60	.21
5. memory inaccuracy (v > u)	10901.5	< .001	.85	.45
6. recalled vs. actual giving (stingy upholders)	1530.5	.15	.14	.06
7. recalled vs. actual giving (gen. upholders)	89.5	.086	.10	-.03
8. recalled vs. actual giving (s.u > g.u)	18585	.047	.24	.09
Static deciders excluded (N=223)				
1. recalled vs. actual giving (all)	6778	.010	.21	.11
2. recalled vs. actual giving (violators)	1473	< .001	.39	.25
3. recalled vs. actual giving (upholders)	1886	.80	.05	.02
4. recalled vs. actual giving (v > u)	4721.5	.008	.39	.21
5. memory inaccuracy (v > u)	5016.5	.045	.30	.16
6. recalled vs. actual giving (stingy upholders)	400.5	.46	.19	.02
7. recalled vs. actual giving (gen. upholders)	557	.75	.07	.03
8. recalled vs. actual giving (s.u > g.u)	2197.5	.75	.26	.03

Supplementary Table 13. Experiment 4a results (with no exclusions, non-givers excluded, and static deciders excluded)

	test statistic	*p*	*d*	**δ**
No exclusions (N=1152)				
1. recalled vs. actual giving (all)	67450.5	< .001	.15	.07
2. recalled vs. actual giving (violators)	18183.5	< .001	.37	.25
3. recalled vs. actual giving (upholders)	15233	.94	.03	-.01
4. recalled vs. actual giving (v > u)	107602	< .001	.36	.23
5. memory inaccuracy (v > u)	94975	< .001	.30	.32
6. recalled vs. actual giving (stingy upholders)	6121	.31	.12	.02
7. recalled vs. actual giving (gen. upholders)	1422	.05	.23	-.18
8. recalled vs. actual giving (s.u > g.u)	15918	.004	.33	.17
Non-givers excluded (N=631)				
1. recalled vs. actual giving (all)	29278	< .001	.18	.09
2. recalled vs. actual giving (violators)	8461.5	< .001	.43	.29
3. recalled vs. actual giving (upholders)	6102.5	.81	.03	-.01
4. recalled vs. actual giving (v > u)	31674	< .001	.45	.27
5. memory inaccuracy (v > u)	26341	< .001	.46	.39
6. recalled vs. actual giving (stingy upholders)	2249	.54	.08	.03
7. recalled vs. actual giving (gen. upholders)	807.5	.71	.10	-.12
8. recalled vs. actual giving (s.u > g.u)	5601	.23	.10	.09
Static deciders excluded (N=385)				
1. recalled vs. actual giving (all)	26861	< .001	.23	.14
2. recalled vs. actual giving (violators)	7986	< .001	.45	.31
3. recalled vs. actual giving (upholders)	5386.5	.97	.02	-.02
4. recalled vs. actual giving (v > u)	13801	< .001	.46	.25
5. memory inaccuracy (v > u)	16604.5	.082	.19	.10
6. recalled vs. actual giving (stingy upholders)	1016	.96	.06	-.03
7. recalled vs. actual giving (gen. upholders)	1597	.94	.01	-.01
8. recalled vs. actual giving (s.u > g.u)	4752	1.00	.04	.00

Supplementary Table 14. Experiment 4b (no responsibility) results (with no exclusions, non-givers excluded, and static deciders excluded)

	test statistic	*p*	*d*	**δ**
No exclusions (N=772)				
1. recalled vs. actual giving (all)	20358.5	.85	.06	<.001
2. recalled vs. actual giving (violators)	4346.5	.49	.13	.01
3. recalled vs. actual giving (upholders)	5913.5	.71	.02	.02
4. recalled vs. actual giving (v > u)	69641.5	.68	.12	.02
5. memory inaccuracy (v > u)	64991	.028	.04	.08
6. recalled vs. actual giving (stingy upholders)	2154.5	.099	.17	.06
7. recalled vs. actual giving (gen. upholders)	635	.025	.23	-.07
8. recalled vs. actual giving (s.u > g.u)	7987.5	.045	.39	.13
Non-givers excluded (N=362)				
1. recalled vs. actual giving (all)	4686	.60	.03	.01
2. recalled vs. actual giving (violators)	819.5	.48	.05	-.04
3. recalled vs. actual giving (upholders)	1600.5	.93	.03	.02
4. recalled vs. actual giving (v > u)	14037	.49	.01	.04
5. memory inaccuracy (v > u)	10093	< .001	.10	.25
6. recalled vs. actual giving (stingy upholders)	615.5	.16	.17	.11
7. recalled vs. actual giving (gen. upholders)	113.5	.025	.36	-.10
8. recalled vs. actual giving (s.u > g.u)	1860.5	.040	.53	.20
Static deciders excluded (N=213)				
1. recalled vs. actual giving (all)	4282.5	.50	.01	<.001
2. recalled vs. actual giving (violators)	819.5	.48	.05	-.05
3. recalled vs. actual giving (upholders)	1375.5	.80	.02	.04
4. recalled vs. actual giving (v > u)	5702	.64	.07	.04
5. memory inaccuracy (v > u)	5030.5	.28	.03	.09
6. recalled vs. actual giving (stingy upholders)	419	.31	.21	.11
7. recalled vs. actual giving (gen. upholders)	205.5	.073	.32	-.05
8. recalled vs. actual giving (s.u > g.u)	1540.5	.12	.52	.16

Supplementary Table 15. Experiment 4b (responsibility) results (with no exclusions, non-givers excluded, and static deciders excluded)

	test statistic	*p*	*d*	**δ**
No exclusions (N=264)				
1. recalled vs. actual giving (all)	7912.5	<.001	.28	.15
2. recalled vs. actual giving (violators)	2918	<.001	.57	.48
3. recalled vs. actual giving (upholders)	1012	.13	.01	-.10
4. recalled vs. actual giving (v > u)	4631	<.001	.67	.46
5. memory inaccuracy (v > u)	5844.5	<.001	.67	.46
6. recalled vs. actual giving (stingy upholders)	327	.62	.26	.00
7. recalled vs. actual giving (gen. upholders)	43	<.001	.59	-.42
8. recalled vs. actual giving (s.u > g.u)	585	.004	.80	.36
Non-givers excluded (N=155)				
1. recalled vs. actual giving (all)	2805	.051	.18	.12
2. recalled vs. actual giving (violators)	101	<.001	.52	.48
3. recalled vs. actual giving (upholders)	372	.066	.08	-.13
4. recalled vs. actual giving (v > u)	1555	<.001	.62	.47
5. memory inaccuracy (v > u)	1974	< .001	.39	.32
6. recalled vs. actual giving (stingy upholders)	122	.90	.17	<.001
7. recalled vs. actual giving (gen. upholders)	16	.003	.66	-.52
8. recalled vs. actual giving (s.u > g.u)	211	.016	.79	.39
Static deciders excluded (N=96)				
1. recalled vs. actual giving (all)	2082	.12	.34	.10
2. recalled vs. actual giving (violators)	831	<.001	.50	.53
3. recalled vs. actual giving (upholders)	225.5	.036	.13	-.27
4. recalled vs. actual giving (v > u)	586	<.001	.61	.49
5. memory inaccuracy (v > u)	955.5	.16	.15	.17
6. recalled vs. actual giving (stingy upholders)	81.5	.50	.34	.10
7. recalled vs. actual giving (gen. upholders)	16	.003	.71	-.57
8. recalled vs. actual giving (s.u > g.u)	107.5	.008	.99	.49

Supplementary References

1. Konow, J. Fair shares: Accountability and cognitive dissonance in allocation decisions. *Am. Econ. Rev.* **90**, 1072–1091 (2000).
2. Babcock, L. & Loewenstein, G. Explaining bargaining impasse: The role of self-serving biases. *J. Econ. Perspect.* **11**, 109–126 (1997).
3. Elliot, A. J. & Devine, P. G. On the motivational nature of cognitive dissonance: Dissonance as psychological discomfort. *J. Pers. Soc. Psychol.* **67**, 382 (1994).
4. Egloff, B., Schmukle, S. C., Burns, L. R., Kohlmann, C.-W. & Hock, M. Facets of dynamic positive affect: differentiating joy, interest, and activation in the positive and negative affect schedule (PANAS). *J. Pers. Soc. Psychol.* **85**, 528 (2003).
5. Barkan, R., Ayal, S. & Ariely, D. Ethical dissonance, justifications, and moral behavior. *Curr. Opin. Psychol.* **6**, 157–161 (2015).
6. Gosling, P., Denizeau, M. & Oberlé, D. Denial of responsibility: a new mode of dissonance reduction. *J. Pers. Soc. Psychol.* **90**, 722 (2006).
7. Moutoussis, M., Dolan, R. J. & Dayan, P. How People Use Social Information to Find out What to Want in the Paradigmatic Case of Inter-temporal Preferences. *PLOS Comput. Biol.* **12**, e1004965 (2016).
8. Fehr, E. & Rangel, A. Neuroeconomic Foundations of Economic Choice–Recent Advances. *J. Econ. Perspect.* **25**, 3–30 (2011).
9. Gneezy, U., Imas, A. & Madarász, K. Conscience accounting: Emotion dynamics and social behavior. *Manag. Sci.* **60**, 2645–2658 (2014).
10. Mullen, E. & Monin, B. Consistency versus licensing effects of past moral behavior. *Annu. Rev. Psychol.* **67**, (2016).
