# Supplementary material for: Motivated misremembering of selfish decisions
Source: Nat Commun. 2020 Apr 29;11:2100. doi: 10.1038/s41467-020-15602-4 (PMC7190661; doi:10.1038/s41467-020-15602-4)
Supplement: Supplementary file 3 — Reporting Summary [file 41467_2020_15602_MOESM3_ESM.pdf]

nature research

Corresponding author(s): Molly J. Crockett
Last updated by author(s): Feb 7, 2020

Reporting Summary

Nature Research wishes to improve the reproducibility of the work that we publish. This form provides structure for consistency and transparency in reporting. For further information on Nature Research policies, see Authors & Referees and the Editorial Policy Checklist.

Statistics

For all statistical analyses, confirm that the following items are present in the figure legend, table legend, main text, or Methods section.

n/a	Confirmed	
☐	☒	The exact sample size (*n*) for each experimental group/condition, given as a discrete number and unit of measurement
☐	☒	A statement on whether measurements were taken from distinct samples or whether the same sample was measured repeatedly
☐	☒	The statistical test(s) used AND whether they are one- or two-sided<br>*Only common tests should be described solely by name; describe more complex techniques in the Methods section.*
☐	☒	A description of all covariates tested
☐	☒	A description of any assumptions or corrections, such as tests of normality and adjustment for multiple comparisons
☐	☒	A full description of the statistical parameters including central tendency (e.g. means) or other basic estimates (e.g. regression coefficient) AND variation (e.g. standard deviation) or associated estimates of uncertainty (e.g. confidence intervals)
☐	☒	For null hypothesis testing, the test statistic (e.g. *F*, *t*, *r*) with confidence intervals, effect sizes, degrees of freedom and *P* value noted<br>*Give P values as exact values whenever suitable.*
☒	☐	For Bayesian analysis, information on the choice of priors and Markov chain Monte Carlo settings
☒	☐	For hierarchical and complex designs, identification of the appropriate level for tests and full reporting of outcomes
☐	☒	Estimates of effect sizes (e.g. Cohen's *d*, Pearson's *r*), indicating how they were calculated

Our web collection on statistics for biologists contains articles on many of the points above.

Software and code

Policy information about availability of computer code

Data collection	Data collection for Experiments 1 and 2 was completed using MATLAB (version 7.12). In Experiments 3, 4a, and 4b, Qualtrics was used.
Data analysis	All data were analyzed using R (version 3.5.0) via RStudio (version 1.0.136).

For manuscripts utilizing custom algorithms or software that are central to the research but not yet described in published literature, software must be made available to editors/reviewers. We strongly encourage code deposition in a community repository (e.g. GitHub). See the Nature Research guidelines for submitting code & software for further information.

Data

Policy information about availability of data

All manuscripts must include a data availability statement. This statement should provide the following information, where applicable:
- Accession codes, unique identifiers, or web links for publicly available datasets
- A list of figures that have associated raw data
- A description of any restrictions on data availability

Raw data and analysis code for all experiments are publicly available in a GitHub repository at https://github.com/carlsonrw/motivated_Mem

Field-specific reporting

Please select the one below that is the best fit for your research. If you are not sure, read the appropriate sections before making your selection.

☐ Life sciences ☒ Behavioural & social sciences ☐ Ecological, evolutionary & environmental sciences

For a reference copy of the document with all sections, see nature.com/documents/nr-reporting-summary-flat.pdf

Behavioural & social sciences study design

All studies must disclose on these points even when the disclosure is negative.

Study description	All five experiments qualify as quantitative, experimental research studies.
Research sample	Experiments 1 and 2 were conducted in a research laboratory. Specifically, we recruited participants from the University of Zürich (UZH) participant database, which primarily consists of undergraduates at UZH (Exp. 1: 112 participants, 57 female, 50 male, 5 did not specify; mean age = 22.0; Exp. 2: 243 participants, 118 female, 125 male; mean age = 22.8). Experiments 3, 4a, and 4b were conducted online. Specifically, we recruited US participants from Amazon Mechanical Turk (Exp. 3: 647 participants, 344 female, 301 male, 2 did not specify; mean age = 36.4; Exp. 4a: 1152 participants, 469 female, 678 male, 5 did not specify; mean age = 34.2; Exp. 4b: 1036 participants, 571 female, 462 male, 3 did not specify; mean age = 35.9. Data collection in Experiments 1 and 2 utilized existing institutional research infrastructure at UZH. Data collection in Experiments 3, 4a, and 4b utilized an online data collection tool (Amazon Mechanical Turk), which offered an effective and efficient resource for running additional experiments to confirm and expand upon our initial lab-based experiments.
Sampling strategy	For Experiments 1 and 2, no power analyses were conducted, however the effect sizes obtained in these experiments informed our power analyses in subsequent experiments. To determine the sample size for Experiments 3, 4a, and 4b, we computed necessary sample sizes using G*Power 3.1.9.3 (see Methods for additional details).
Data collection	Data collection for Experiments 1 and 2 was conducted in a laboratory using MATLAB. Responses where made using a standard computer mouse and keyboard. Data collection for all subsequent experiments was conducted online, with participants recruited from Amazon Mechanical Turk.
Timing	Experiment 1: October 2011 - December 2011, Experiment 2: April 2012 - June 2012, Experiment 3: October 2017, Experiment 4a: June 2019, Experiment 4b: July 2019
Data exclusions	We excluded participants if (i) they failed an attention check, (ii) they were an extreme outlier (i.e., > 4 standard deviations from the mean) on our key dependent variable, or (iii) they reported suspicion about key aspects of the experiment. We report our analysis with all participants included in the Supplemental Information, and these results are consistent with those reported in the main text.
Non-participation	No dropouts were recorded.
Randomization	In Experiment 4b, participants were randomly assigned to experimental conditions.

Reporting for specific materials, systems and methods

We require information from authors about some types of materials, experimental systems and methods used in many studies. Here, indicate whether each material, system or method listed is relevant to your study. If you are not sure if a list item applies to your research, read the appropriate section before selecting a response.

Materials & experimental systems

n/a	Involved in the study	
☒	☐	Antibodies
☒	☐	Eukaryotic cell lines
☒	☐	Palaeontology
☒	☐	Animals and other organisms
☐	☒	Human research participants
☒	☐	Clinical data

Methods

n/a	Involved in the study	
☒	☐	ChIP-seq
☒	☐	Flow cytometry
☒	☐	MRI-based neuroimaging

Human research participants

Policy information about studies involving human research participants

Population characteristics	See above
Recruitment	Participants were recruited from the participant pools specified above through nondescript advertisements for an experiment about social behavior
Ethics oversight	University of Zurich Ethics Commission; Yale University Human Subjects Committee

Note that full information on the approval of the study protocol must also be provided in the manuscript.
